# Supplementary material for: Consensus assessment of the contamination level of publicly available cyanobacterial genomes
Source: PLoS One. 2018 Jul 25;13(7):e0200323. doi: 10.1371/journal.pone.0200323 (PMC6059444; doi:10.1371/journal.pone.0200323)
Supplement: S1 Appendix — (PDF) [file pone.0200323.s001.pdf]

Supplemental Note 1: Optimization of Kraken database

Definition of the confidence threshold

During database construction, Kraken labels each unique kmer with its containing genome or with the LCA if a given kmer is found in more than one genome. When querying a sequence, each kmer of the query sequence is mapped to the corresponding LCA in the database. This series of taxa is further mapped on the nodes of the taxonomic tree, then weights (number of kmers) are associated to these nodes, and the query is globally labelled according to the higher scoring path in the tree (for details, see Figure 1 in Wood & Salzberg [1]). The precision (taxonomic rank in the lineage of an organism) of the labelling can be fine-tuned through a parameter called the confidence threshold. The latter is defined as a ratio of kmers and allows users to adjust Kraken precision by pruning the subtree based on the weight of the nodes: the higher the confidence threshold, the higher the number of kmers required for a node to label a sequence, leading to higher taxonomic-rank classifications and thus a lower precision (e.g., at the family level instead of the genus level). Sequences that do not share enough kmers with those in the database are labelled as unclassified. Given its relative complexity, we advise the reader to refer to Wood & Salzberg [1] for the details of Kraken algorithm.

Choice of the kmer size

Kraken analyzes genomes sequence by sequence. To increase the resolution, we split cyanobacterial genome assemblies into (non-overlapping) pseudo-reads of 250 nt using a custom Perl script. This step ensures that even small parts of a genome sequence can influence the results, thereby avoiding a loss of precision because of too highly-ranked LCA labels. We analyzed cyanobacterial pseudo-reads against the default genome set (provided with Kraken) using three different sizes of kmer: 21, 25 and 31 (default sizes). Since kmer size is fixed at database construction, it is a property of the database. We further tested six different confidence thresholds (0.00, 0.02, 0.04, 0.06, 0.08, 0.10). S2 Fig shows the effect of these two parameters on Kraken sensitivity, expressed as the fraction of the pseudo-reads of 250 nt classified for each assembly. Except for the smallest kmer size (21) and the default confidence threshold (0.00), the interquartile range (IQR) of sensitivity is always large (>40%). Regardless of the confidence threshold, Kraken classified a higher fraction of the assemblies at a kmer size of 21, suggesting that it is the most adapted to our dataset. At this kmer size, the medians of the overall sensitivity slightly decreased from 97% (at 0.00) to 85% (at 0.10) when increasing the confidence threshold. In contrast, the confidence threshold had a strong effect on the IQR of the fractions of classified sequences, indicating that, for a number of organisms, sensitivity drops dramatically as the confidence threshold is raised.

The need for a larger set of reference genomes

We first estimated the contamination level of cyanobacterial genome assemblies using the default Kraken database, based on a set of 7025 bacterial strains (of which 71 are Cyanobacteria). Owing to its strategy based on signature kmers, Kraken can only identify what it already knows. Hence, genomes that are evolutionarily distant from all the genomes used to build the database are expected to have a

high fraction of original kmers and a correspondingly high fraction of unclassified sequences. This hypothesized bias was confirmed when comparing the fraction of classified sequences (confidence = 0.04) in every assembly to the genetic distance separating an assembly from its closest relative in the database, as estimated by ML on predicted SSU rRNA (16S) genes (S3a Fig). From this analysis, it is clear that genome assemblies without close relatives in the reference genome database have a lower fraction of classified sequences. This solid negative linear correlation (Pearson r = -0.89) was extremely significant (P-value = 3.24e-94). To address this problem and improve the classification of distant strains, we built a new Kraken database using a larger set of genomes based on the release 30 of Ensembl Bacteria (EB30).

Curation of the reference database

Kraken results can be strongly affected by contaminated or chimerical genomes included in its reference database [1]. For instance, when using the full set of genomes (29,320) in EB30, the genome of *Synechocystis* PCC 6803 [2] does not appear to contain >10% of cyanobacterial sequences. This surprising result is caused by the inclusion in EB30 of *Bacillus subtilis* BEST 7613 [3,4], which is an artificial chimera between a *Bacillus* and a *Synechocystis*. Hence, as nearly all the kmers of *Synechocystis* are (wrongly) shared with *Bacillus,* Kraken database only labels them as “Bacteria”, the last common ancestor (LCA) of the two strains, which later on prevents these *Synechocystis* kmers to be correctly classified as cyanobacterial kmers. To remove contaminated and chimerical genomes from EB30, we took advantage of the LCA classification performed by Kraken during the database construction step. Briefly, after running Kraken on all 29,320 EB30 genomes against a preliminary database made of the very same genomes (*all-vs-all*), we eliminated those genomes that showed sudden drops (>10%) in classification percentage between successive taxonomic ranks of their lineage. For example, a genome with 99% match to Bacteria but only 51% match to Firmicutes was eliminated because nearly 48% of its kmers could not be identified with enough precision. Such events are due to the fact that, in the database, these kmers are only labelled with a high-ranking taxon, “Bacteria” in this case. Since this indicates that these kmers are (anomalously) shared by very distant organisms, we conservatively chose to exclude the corresponding genomes, even if some of these genomes might have been actually non-contaminated but very distantly related to those contained in Kraken database. Hence, we eliminated 1558 genomes from the genome set of EB30 and used the 27,762 remaining genomes to build a curated database with a kmer size of 21.

For our study, we deliberately retained all 170 cyanobacterial genomes from EB30 in the curated database, even those that our preliminary tests flagged as contaminated (i.e., GCA_000817785.1, GCA_000817735.1, GCA_000828085.1, GCA_000828075.1 and GCA_000153045.1). Our reasoning was that the genuine cyanobacterial kmers introduced by these genomes would significantly improve the classification of cyanobacterial assemblies, whereas their less abundant non-cyanobacterial kmers would be overwhelmed by those provided by the thousands of other reference bacterial genomes. As shown in S3b Fig, the new database indeed improved the classification of cyanobacterial assemblies, Kraken sensitivity being twice less affected by an increase of the genetic distance (slope rising from -850 to -419, Pearson r = -0.93, P-value = 4.41e-78). When comparing the contamination level estimates obtained for 238 non-reference cyanobacterial assemblies analyzed with either the default Kraken database or our curated database, estimates appeared highly correlated (Pearson r = 0.87, P-value = 9.63e-76), and only 31 assemblies showed contamination levels (marginally) reduced with the latter database (mean reduction: 0.21%), which confirmed our expectations.

The direct download links to the assemblies in the curated database are available at https://figshare.com/s/bdcc314a7b90b00c1274. For the published version, the five contaminated cyanobacterial genome assemblies have been commented out.

References

1. Wood DE, Salzberg SL. Kraken: ultrafast metagenomic sequence classification using exact alignments. Genome Biol. 2014;15: R46. doi:10.1186/gb-2014-15-3-r46

2. Trautmann D, Voß B, Wilde A, Al-Babili S, Hess WR. Microevolution in Cyanobacteria: Re-sequencing a Motile Substrain of Synechocystis sp. PCC 6803. DNA Res. 2012; dss024. doi:10.1093/dnares/dss024

3. Itaya M, Tsuge K, Koizumi M, Fujita K. Combining two genomes in one cell: Stable cloning of the Synechocystis PCC6803 genome in the Bacillus subtilis 168 genome. Proc Natl Acad Sci U S A. 2005;102: 15971–15976. doi:10.1073/pnas.0503868102

4. Watanabe S, Shiwa Y, Itaya M, Yoshikawa H. Complete Sequence of the First Chimera Genome Constructed by Cloning the Whole Genome of Synechocystis Strain PCC6803 into the Bacillus subtilis 168 Genome. J Bacteriol. 2012;194: 7007–7007. doi:10.1128/JB.01798-12
