# Supplementary figures and images for: Consensus assessment of the contamination level of publicly available cyanobacterial genomes
Source: PLoS One. 2018 Jul 25;13(7):e0200323. doi: 10.1371/journal.pone.0200323 (PMC6059444; doi:10.1371/journal.pone.0200323)

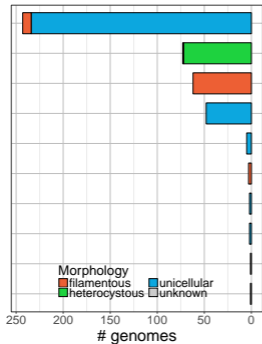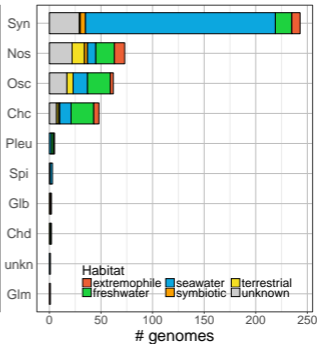

Supplement: S1 Fig — The 440 strains were classified into the eight orders defined in Komarek et al. 2014 [Syn: Synechococcales, Nos: Nostocales, Osc: Oscillatoriales, Chc: Chroococcales, Pleu: Pleurocapsales, Spi: Spirulinales, Glb: Gloeobacterales, Chd: Chroococcidiopsidales (and Glm: Gloeoemargaritales)], and further broken into either four morphologies (left panel: unicellular, filamentous, heterocystous, unknown) or six habitats (right panel: seawater, freshwater, terrestrial, extremophile, symbiotic and unknown). Horizontal axes are in number of assemblies. (PDF) [file pone.0200323.s002.pdf]

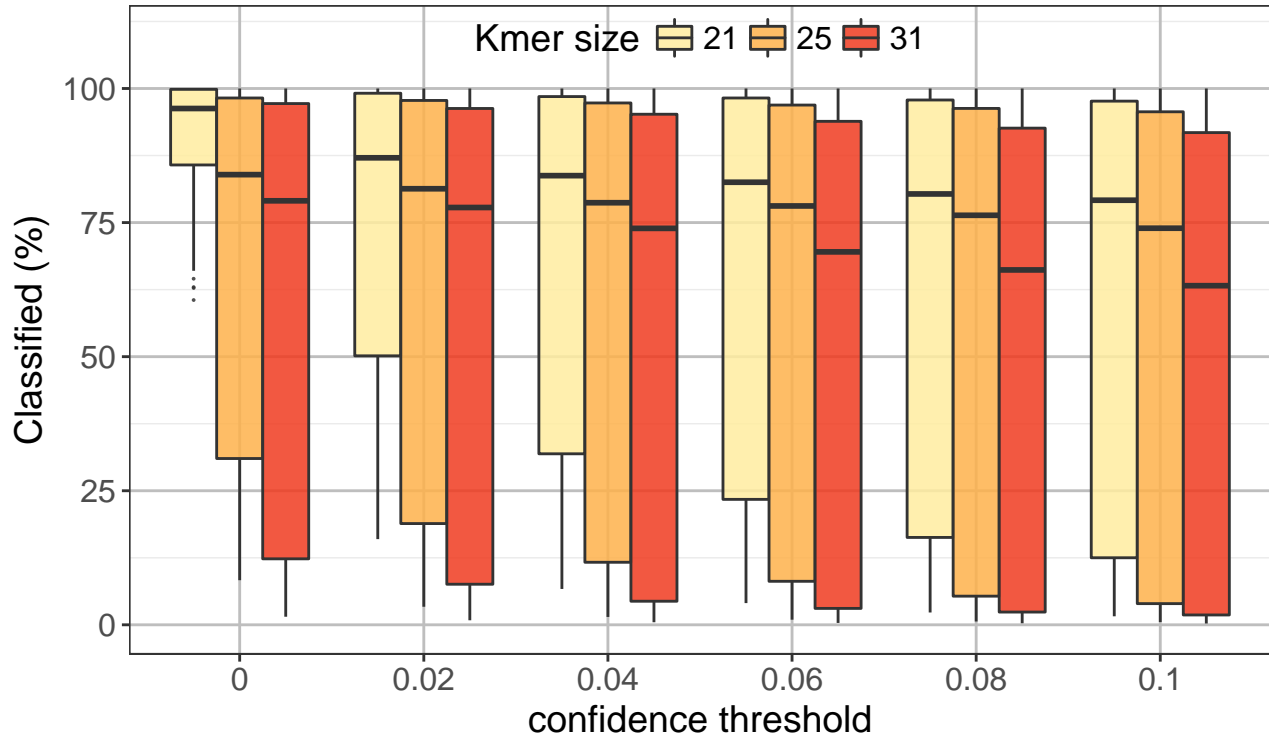

Supplement: S2 Fig — The classified fractions of the 440 cyanobacterial genomes (expressed in %) are summarized as one box-and-whiskers plot for every combination of Kraken parameters. Boxes correspond to interquartile ranges (IQR = Q3–Q1), whereas medians (Q2) are shown as thick horizontal black lines. Upper and lower whiskers extend from the hinge to the largest and lowest value no further than 1.5 * IQR from the hinge, respectively. Data points beyond the end of the whiskers are outliers and are plotted individually. (PDF) [file pone.0200323.s003.pdf]

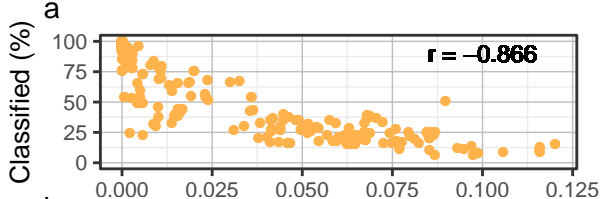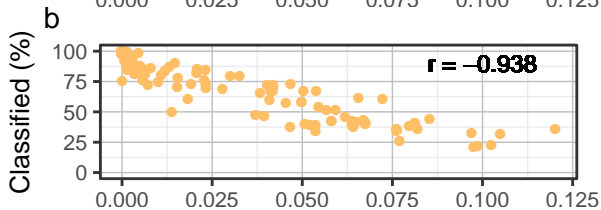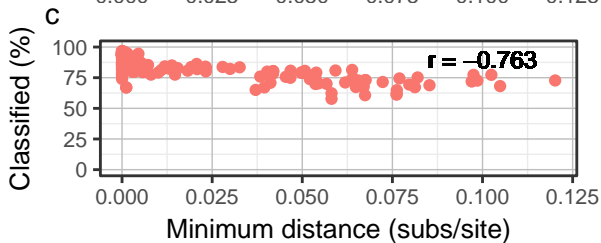

Supplement: S3 Fig — For each of the 343 cyanobacterial genome assemblies, the classified fraction using Kraken (at a kmer size of 21, expressed in %) (a,b) or DIAMOND blastx (c) is plotted against its evolutionary distance (expressed in substitutions per site) to the most closely related genome in the default Kraken database (a) or in the curated Ensembl 30 database (b,c). Distances were estimated by predicting and comparing SSU rRNA (16S) genes under the GTR+Γ4 model, and the minimum distance was selected for every genome of our dataset. r is the Pearson correlation coefficient between the two variables. (PDF) [file pone.0200323.s004.pdf]

Cyanobacterial genome assemblies

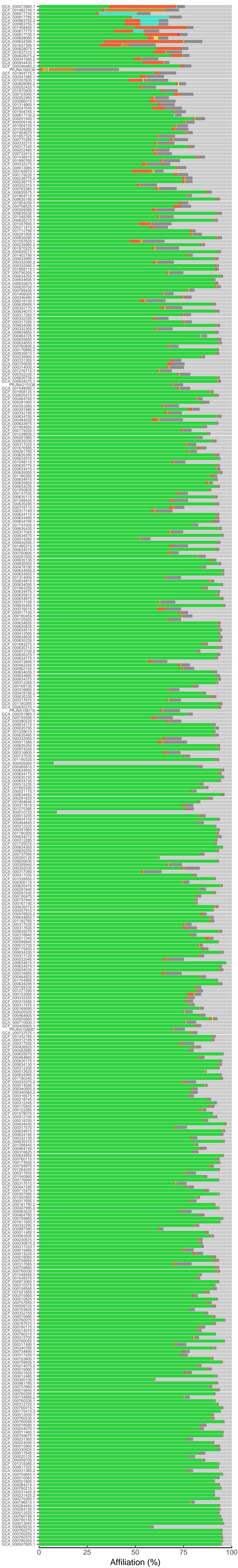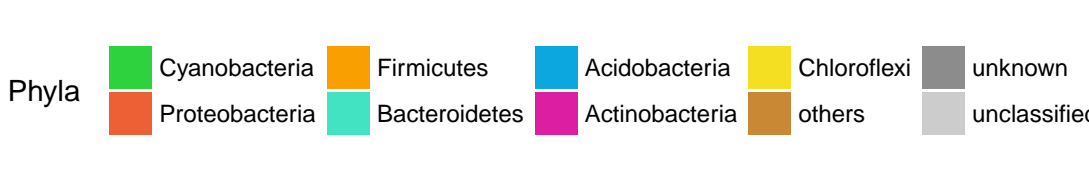

Supplement: S5 Fig — The 440 assemblies (in the global ranking order of S2 Table) were analyzed with DIAMOND blastx, as explained in the main text (using a LCA approach against a protein version of our curated Ensembl 30 database). Taxonomic classifications (expressed in % of the genomic sequence) are summarized at the phylum level. The “unclassified” classification corresponds to sequences that do not match any reference protein in the database, whereas “unknown” corresponds to high-ranking LCAs (Bacteria or Terrabacteria). “others” include the following phyla (in descending order of frequency): Euryarchaeota, Nitrospinae, Deinococcus-Thermus, Candidatus Tectomicrobia, Verrucomicrobia, Spirochaetes, Nitrospirae, Armatimonadetes, Aquificae, Synergistetes, Gemmatimonadetes, Chlamydiae, Thermotogae, Fusobacteria, Deferribacteres, Thaumarchaeota, Thermodesulfobacteria, Tenericutes, Chrysiogenetes, Crenarchaeota, Lentisphaerae, Caldiserica, Dictyoglomi. (PDF) [file pone.0200323.s006.pdf]
