# Supplementary material for: Consensus assessment of the contamination level of publicly available cyanobacterial genomes
Source: PLoS One. 2018 Jul 25;13(7):e0200323. doi: 10.1371/journal.pone.0200323 (PMC6059444; doi:10.1371/journal.pone.0200323)
Supplement: S4 Fig — The top-21 ranking assemblies were split into segments of 10,000 nt for detailed analysis. On the X axis, segment-containing scaffolds were sorted by increasing average GC-content, with vertical thin lines representing scaffold boundaries. Segments were classified as either cyanobacterial (green), contaminated (red), unknown (grey) or unclassified (white), using DIAMOND blastx. The overlaid light blue curve is the GC-content of the corresponding segments. Open symbols give the locations of the detected ribosomal genes (circles: SSU rRNA (16S), squares: ribosomal proteins), either from cyanobacterial sources (green) or from non-cyanobacterial sources (red). (PDF) [file pone.0200323.s005.pdf]

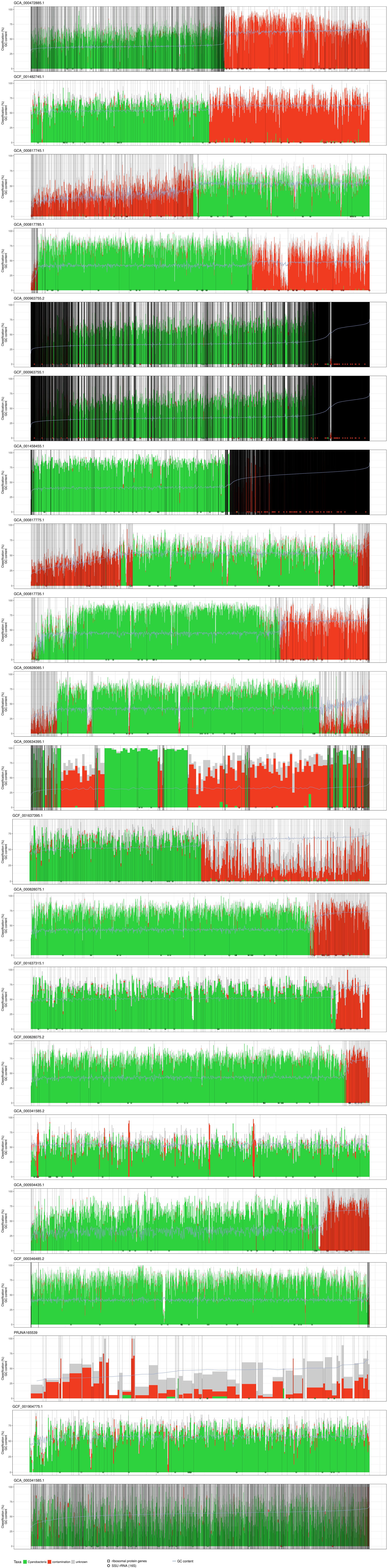
GCA_000472885.1
GCF_001482745.1
GCA_000817745.1
GCA_000817785.1
GCA_000963755.2
GCF_000963755.1
GCA_001458455.1
GCA_000817775.1
GCA_000817735.1
GCA_000828085.1
GCA_000634395.1
GCF_001637395.1
GCA_000828075.1
GCF_001637315.1
GCF_000828075.2
GCA_000341585.2
GCA_000934435.1
GCF_000346485.2
PRJNA165539
GCF_001904775.1
GCA_000341585.1
Classification (%)
GC content
0
25
50
75
100
Taxa
Cyanobacteria
contamination
unknown
ribosomal protein genes
SSU rRNA (16S)
GC content
